# Sodium-glucose cotransporter-2 inhibitors (SGLT2) in frail or older people with type 2 diabetes and heart failure: a systematic review and meta-analysis

**DOI:** 10.1093/ageing/afad254

**Published:** 2024-01-29

**Authors:** Rami Aldafas, Tomas Crabtree, Mohammed Alkharaiji, Yana Vinogradova, Iskandar Idris

**Affiliations:** Division of Graduate Entry Medicine and Health Sciences, University of Nottingham, Derby, UK; Faculty of Public Health, College of Health Science, The Saudi Electronic University, Riyadh, Saudi Arabia; Division of Graduate Entry Medicine and Health Sciences, University of Nottingham, Derby, UK; Department of Endocrinology and Diabetes, University Hospitals Derby and Burton NHS Foundation Trust, Derby, UK; Faculty of Public Health, College of Health Science, The Saudi Electronic University, Riyadh, Saudi Arabia; Division of Primary Care, University of Nottingham, Nottingham NG2 7RD, UK; Division of Graduate Entry Medicine and Health Sciences, University of Nottingham, Derby, UK; Department of Endocrinology and Diabetes, University Hospitals Derby and Burton NHS Foundation Trust, Derby, UK; MRC-Versus Arthritis Centre for Musculoskeletal Ageing Research, NIHR, Nottingham BRC, University of Nottingham, Derby, UK

**Keywords:** sodium-glucose cotransporter 2 inhibitors, frail, older, type 2 diabetes, heart failure, systematic review, older people

## Abstract

**Objective:**

Sodium-glucose cotransporter-2 inhibitors (SGLT2Is) reduce cardio-metabolic and renal outcomes in patients with type 2 diabetes (T2D) but their efficacy and safety in older or frail individuals remains unclear.

**Methods:**

We searched PubMed, Scopus, Web of Science, Cochrane CENTRA and Google Scholar and selected randomised controlled trials and observational studies comparing SGLT2Is versus placebo/other glucose-lowering agent for people with frailty or older individuals (>65 years) with T2D and heart failure (HF). Extracted data on the change in HbA1c % and safety outcomes were pooled in a random-effects meta-analysis model.

**Results:**

We included data from 20 studies (22 reports; *N* = 77,083 patients). SGLT2Is did not significantly reduce HbA1c level (mean difference −0.13, 95%CI: −0.41 to 0.14). SGLT2Is were associated with a significant reduction in the risk of all-cause mortality (risk ratio (RR) 0.81, 95%CI: −0.69 to 0.95), cardiac death (RR 0.80, 95%CI: −0.94 to 0.69) and hospitalisation for heart failure (HHF) (RR 0.69, 95%CI: 0.59–0.81). However, SGLT2Is did not demonstrate significant effect in reducing in the risk of macrovascular events (acute coronary syndrome or cerebral vascular occlusion), renal progression/composite renal endpoint, acute kidney injury, worsening HF, atrial fibrillation or diabetic ketoacidosis.

**Conclusions:**

In older or frail patients with T2D and HF, SGLT2Is are consistently linked with a decrease in total mortality and the overall burden of cardiovascular (CV) events, including HHF events and cardiac death, but not protective for macrovascular death or renal events. Adverse events were more difficult to quantify but the risk of diabetic ketoacidosis or acute kidney injury was not significantly increase.

## Key Points

- Frailty is highly prevalent in people with type 2 diabetes.
- Despite well recognised cardioprotective benefits of SGLT2 inhibitor, its effectiveness in people with type 2 diabetes with heart failure remains who are frail remains unclear.
- Further evidence is required to support he use of SGLT2i in the older and frail population.
- Our study showed that in older and frail patients with type 2 diabetes and heart failure, SGLT inhibitor are associated with a reduction in total mortality, heart failure events and cardiac death, but not protective for macrovascular death or renal events.
- Adverse events within this patient group remains difficult to quantify.

## Introduction

Type 2 diabetes mellitus (T2DM) is an independent contributor to the increased risk of heart failure (HF), cardiovascular (CV) risk, impaired renal function and premature death [1–4]. Diabetes prevalence rises with age, peaking at 24% in the 75–79 age group. In 2019, 135.6 million people aged 65–99 had diabetes and expected to reach 276.2 million by 2045, with most cases in low- and middle-income countries [5].

Frailty is a multi-dimensional concept characterised by decreased physiological reserve and increased vulnerability to adverse health outcomes. It can manifest as weakness, fatigue, reduced physical activity and increased susceptibility to illness or disability [6, 7]. T2DM has been shown to accelerate the onset of frailty, in addition to the established DM-related vascular complications [8]. The dynamic nature of frailty increases vulnerability to adverse health outcomes, prompting various international clinical guidelines to underscore its significance among older (aged ≥65 years) or frail patients with T2DM and HF [9–11].

Sodium-glucose co-transporter 2 (SGLT2) inhibitors function in the proximal renal tubule to interrupt Na and glucose reabsorption and are increasingly used in routine clinical practice [12]. Numerous landmark clinical trials in people with T2DM have now confirmed the irrefutable benefits of SGLT2 inhibitors for reducing mortality and CV risk as well as preventing both incident or recurrent hospitalisation for heart failure (HHF) and declining renal function [13–21]. Additional studies have also shown cardio-renal benefits of SGLT2 inhibitors independent of diabetes status [16, 22–24].

Despite the conclusive benefits of SGLT2 inhibitors in patients with T2DM and HF, their efficacy and risk-benefits profile in older individuals and those with frailty remain a matter of ongoing debate and have not been systematically investigated. Recent meta-analyses [25] have showed substantial benefits of SGLT2 inhibitors, including a reduction in HHF and a slowing of chronic kidney disease progression in older adults, with or without T2DM. Despite this, use of SGLT2 inhibitors in older adults remains a concern due to the potential risk of acute kidney injury (AKI) or diabetic ketoacidosis (DKA) [26–29], partially due to its effect on volume depletion.

Further evidence is therefore needed to support the use of SGLT2 inhibitors in the older or frail population with T2DM. The updated NICE T2DM clinical guideline now calls for their broader use, alongside metformin, among patients with HF or at high risks of CV disease irrespective of HbA1c levels [30]. Current frailty-specific guidelines for T2DM also emphasise a need to individualised management, in which SGLT2 inhibitors may provide cardioprotective effects on frail patients with co-morbidities [31].

We therefore aimed to analyse the clinical efficacy and safety of SGLT2 inhibitors in people with frailty or older people (aged ≥65 years) with T2DM and HF.

## Materials and methods

We performed this systematic review and meta-analysis based on the preferred reporting items for systematic review and meta-analysis (PRISMA) statement [32] and according to the Cochrane Handbook [33].

### Literature search strategy

We performed a comprehensive literature search of five electronic databases (PubMed, Scopus, Web of Science, Cochrane CENTRAL, Google Scholar) through February 2023, using these search keywords ‘Sodium-glucose co-transporter-2 inhibitors’, ‘SGLT2 inhibitors’, ‘canagliflozin’, ‘dapagliflozin’, ‘empagliflozin’, ‘ipragliflozin’, ‘sotagliflozin’, ‘luseogliflozin’, ‘*Gliflozin’, ‘Heart failure’, ‘heart right ventricle failure’, ‘congenital heart failure’, ‘Diabetes Mellitus’, ‘type 2 diabetes’, ‘T2DM’, ‘T2DM’, ‘non-insulin dependent’, ‘NIDDM’, ‘Frail’, ‘frailty’, ‘Elderly’, ‘Older’ and ‘Old’. These keywords were amalgamated using Boolean operators for each database, as appropriate. We refrained from applying any filters except for language. All duplicates were removed using EndNote software, and all references to the included studies were screened manually for any eligible studies.

### Study selection

Two independent authors (R.A. and T.C.) systematically screened the titles and abstracts of identified records. Following this, they compared their results, reached a consensus on which studies met the initial inclusion criteria and proceeded to conduct full-text screening for the selected studies. Only relevant original research encompassing randomised controlled trials (RCTs) and observational studies reported in full-text that compared SGLT2 inhibitors with either placebo or any other glucose-lowering agent was selected. We adopted a comprehensive approach to select studies that met the predefined criteria and included studies that used or did not use propensity score matching (PS matching). Our target population are individuals aged 65 years and older with both HF and T2D. We excluded single-arm studies, animal studies and studies reported in a language other than English (due to practical considerations related to language accessibility and resource constraints). A third author (I.I.) adjudicated any discrepancies between authors.

### Risk of bias assessment

Independently, two reviewers used the Cochrane risk of bias (ROB) tool to assess the methodological quality [34] for the included RCTs. We evaluated the risk of bias across various domains, including sequence generation (indicating selection bias), allocation sequence concealment (reflecting selection bias), blinding of participants and personnel (performance bias), blinding of outcome assessment (detection bias), incomplete outcome data (attrition bias), selective outcome reporting (reporting bias) and other potential sources of bias. Our assessments are categorised as either 'Low risk', 'High risk' or 'Unclear risk' of bias. For this evaluation, we referred to the quality assessment table found in (part 2, Chapter 8.5) of the Cochrane handbook of systematic reviews of interventions. For the observational studies, the Newcastle–Ottawa Scale (NOS) was used [35]. Each study received a star rating ranging from 0 to 9 based on three criteria: S for selection (0–4 stars), C for comparability (0–2 stars) and O for outcome (0–3 stars). Studies receiving six or more stars were categorised as good quality [36]. In case of disagreement among reviewers during the risk of bias assessment, a consensus is sought through discussion. If consensus cannot be reached, an independent third reviewer is consulted for an objective assessment to resolve the disagreement.

### Data extraction and study outcomes

The lead author prepared formatted Excel sheets including demographic data and study characteristics, ROB assessment and outcomes of interest.

The data extraction process was conducted independently by two authors, followed by a double-checking step to ensure accuracy and consistency. The review authors extracted the following data: demographic data, glycated haemoglobin (HbA1c) and safety profile (CV and renal outcomes). These studies excluded patients with type 1 diabetes due to increased risks of DKA. Any incomplete or incompatible data have been dealt with through methods recommended in the Cochrane Handbook [37].

### Statistical analysis

For continuous variables (change in HbA1c level), the mean difference (MD) and its 95% confidence interval (CI) were calculated, while the risk ratios (RRs) with 95% CI were calculated for dichotomous variables (safety outcomes). The statistical model used was the generic inverse variance weighting methodology. The statistical analysis was performed using Review Manager (RevMan) software version 5.4. To calculate the MD and standard error (SE) for the change in HbA1c levels, we utilised the RevMan calculator. These values were then used to derive the overall MD and a corresponding 95% CI. The included studies were of different populations (i.e. geographical location, demographic characteristics and medical conditions) due to different interventions across the studies. Therefore, we used a random-effect model (DerSimonian–Laird) as we could not rely on each study as the true effect size of the drug evaluated [33, 38]. We also assessed the degree of Heterogeneity between studies using *I^2^* statistics. Ranges of 0–24, 25–74 and 75–100% were used to define low, moderate and high heterogeneity, respectively [33]. We evaluated publication bias using funnel plots and conducted the Egger test to assess both funnel plot asymmetry and the presence of publication bias [39]. Further analysis based on the study design is also performed. Leave-one-out sensitivity analyses were performed to assess the impact of excluding individual studies on the summary findings and heterogeneity in relation to the change in HbA1c. These analyses helped determine the extent to which a particular study influences the overall effect size and the level of variation observed among studies. To account for the variation observed in studies with a wide standard deviation (SD) but a mean and median age of 65, a subgroup analysis was conducted.

**Figure 1:**
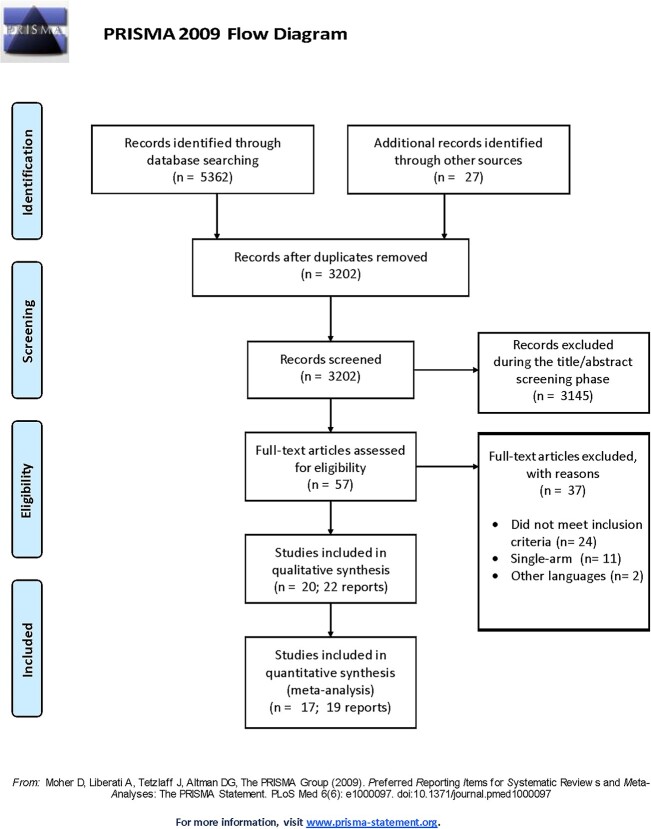
The flow diagram of the study selection procedure. *The reasons for exclusion during this phase included records that were irrelevant to the research topic, did not involve the specified patient population or were not original research articles.*

**Table 1:** Characteristics of the included studies

Study ID	Country	Study design	N. of patients	Study groups	Study duration/follow-up	N. of patients in each group	Age, mean ± SD	Gender (male), *n*	Weight, mean ± SD	LVEF (%), mean ± SD	Baseline HbA1c mean ± SD
Akasaka et al. 2022 (EXCEED study) [1]	Japan	RCT, open-label, multicentre	68	Ipragliflozin	24 weeks	36	71.9 ± 8.0	22	63.3 ± 11.2	60.9 ± 7.0	8.1 ± 1.0
				Conventional treatment		32	70.3 ± 8.5	19	66.9 ± 15.5	60.4 ± 8.2	7.9 ± 1.1
de Boer et al. 2020 [2]	55 centres across 21 countries	RCT, multicentre, parallel-group phase IIA	124	Licogliflozin 2.5 mg	12 weeks	15	Median 70.0 (range 62.0–75.0)	14	Median 99.0 (range 77.5–114.9)	<45%: 5 (33.3%) ≥45%: 10 (66.7%)	—
				Licogliflozin 10 mg		16	Median 72.5 (range 66.0–75.5)	12	Median 90.0 (range 78.8–106.2)	<45%: 4 (25.0%) ≥45%: 12 (75.0%)	—
				Licogliflozin 50 mg		30	Median 66 (range 60.0–71.0)	24	Median 94.0 (range 87.0–104.2)	<45%: 6 (20.0%) ≥45%: 24 (80.0%)	—
				Empagliflozin 25 mg		30	Median 68.5 (range 62.0–74.0)	20	Median 87.4 (range 76.0–107.4)	<45%: 7 (23.3%) ≥45%: 23 (76.7%)	—
				Placebo		33	Median 71 (range 59.0–74.0)	19	Median 87.3 (range 79.0–97.2)	<45%: 8 (24.2%) ≥45%: 25 (75.8%)	—
Weng et al. 2023 [3]	Taiwan	Retrospective cohort study (2016–2018)	366	SGLT2i	From 2016 to 2018; Terminated on 31 December 2019	183	65.09 ± 13.24	121	—	—	7.96 ± 1.88
				Non-SGLT2i		183	65.36 ± 12.69	122	—	—	7.63 ± 1.83
Becher et al. 2021 [4]	Sweden (Swedish HF Registry (SwedeHF)	Observational	1,444	SGLT2i	Between 1 January 2016 and 31 December 2018	361	Median 69.0 (range 62.0–75.0)	283	—	—	—
				Non-SGLT2i		1,083	Median 70.0 (range 64.0–74.0)	856	—	—	—
Ejiri et al. 2020 [5]	Japan	RCT, open-label, multicentre	165	Luseogliflozin	24 weeks	83	71.7 ± 7.7	55	64.6 ± 12.7	57 ± 9.4	7.0 ± 0.7
				Voglibose		82	74.6 ± 7.7	48	63.5 ± 13.1	58 ± 9.4	6.9 ± 0.8
Filippatos et al. 2022 [6]	EMPEROR-Preserved [622 centres in 23 countries]	Secondary analysis of EMPEROR-Preserved	1,466	Empagliflozin + Diabetes Placebo + Diabetes	36 months	1,466	70.9 ± 9.0	1,682	—	53.9 ± 8.7	7.26 ± 1.50
Lee et al. 2021 (SUGAR-DM-HF trial) [7]	Scotland	RCT, multicentre, double-blind	105	Empagliflozin	36 weeks	52	68.2 ± 11.7	34	(85.2 ± 19.1) of 44	—	7.5 ± 1.6
	Placebo					53	69.2 ± 10.6	43	(86.5 ± 15.2) of 50	—	7.0 ± 1.4
Li et al. 2022 [8]	Montefiore Medical Center, United States	Retrospective Observational Cohort study	250	SGLT2i	295 days	89	68 ± 12.2	31	—	—	8.6 ± 1.4
				Sitagliptin		161	69 ± 13.3	55	—	—	8.8 ± 2.1
Martin et al. 2021 [9]	University Reina Sofía Hospital, Córdoba, Spain	Cohort study	102	Canagliflozin	22 months	45	69 ± 10	30	—	45.4 ± 17.9	7.4 ± 1.5
				Control		57	73 ± 11	27	—	49.9 ± 17.8	6.8 ± 2.5
Perez-Belmonte et al. 2021 [10]	4 hospitals in Málaga, Spain	Observational study	182	Empagliflozin	Between 2017 and 2020	91	72.0 ± 5.6	45	90.0 ± 10.9	46.8 ± 24.1	7.2 ± 0.6
				basal-bolus insulin regimen		91	72.7 ± 5.8	43	89.4 ± 8.6	47.0 ± 23.0	7.1 ± 0.5
Perez-Belmonte et al. 2022 [11]		Observational study	158	Empagliflozin	Between September 2015 and June 2021	79	85.0 ± 4.1	40	90.4 ± 10.4	46.5 ± 22.9	7.5 ± 0.9
				basal-bolus insulin regimen		79	85.2 ± 4.4	38	89.2 ± 8.0	47.0 ± 23.5	7.4 ± 0.8
Petrie et al. 2020 [12]	410 sites in 20 countries	RCT, double-blind	2,139	Dapagliflozin	Between 15 February 2017 and 17 August 2018, with final follow-up on 6 June 2019	1,075	66.3 ± 9.9	835	—	31.4 ± 6.6	7.4 ± 1.5
				Placebo		1,064	66.7 ± 9.8	827	—	31.0 ± 6.8	7.4 ± 1.6
Singh et al. 2020 (REFORM Trial) [13]	NHS tayside, Scotland	RCT, single centre	56	Dapagliflozin	1 year	28	66.9 ± 7.0	18	92.9	44.5 ± 12.4	63.0 ± 17.8
				Placebo		28	67.4 ± 6.8	19	90.2	46.5 ± 11.7	58.6 ± 16.4
Tamaki et al. 2021 [14]	Japan	RCT, Prospective	59	Empagliflozin	Between January 2017 and February 2020	30	Median 80 (range 77–83)	18	—	Median 44 (range 32–61)	Median 6.9 (range 6.4–7.6)
				Conventional		29	Median 82 (range 75–84)	18	—	Median 39 (range 32–51)	Median 7.4 (range 6.8–8.4)
Tanaka et al. 2020 (CANDLE) [15]	34 centres in Japan	RCT, multicentre	233	Canagliflozin	24 weeks	113	68.3 ± 9.8	88	66.6 ± 12.7	(57.6 ± 14.6) of 101	(6.9 ± 0.7) of 112
				Glimepiride		120	68.9 ± 10.4	86	66.4 ± 13.6	(57.7 ± 14.2) of 111	(7.1 ± 0.9) of 117
Ueda et al. 2021 (the CANONICAL study) [16]	Japan	RCT, multicentre	82	Canagliflozin	24 weeks	42	76.5 ± 6.4	28	62.89 ± 10.96	61.1 ± 7.8	7.13 ± 0.74
				Standard diabetes treatment		40	75.9 ± 5.8	27	63.90 ± 11.38	61.9 ± 7.6	6.90 ± 0.55
Szarek et al. 2021 [17]	306 sites in 32 countries.	RCT, double-blind, placebo-controlled	1,222	Sotagliflozin	9.0 months (interquartile range, 4.9–13.2 months)	608	Median 69 (range 63–76)	410	—	481 ± 79.1	—
				Placebo		614	Median 70 (range 64–76)	400	—	485 ± 79.0	—
Desai et al. 2022 (cohort 1) [18]	United States	Observational study	22,858	Empagliflozin	12 months	11,429	71.85 ± 5.02	6,054	—	—	—
				Sitagliptin		11,429	71.85 ± 5.023	6,045	—	—	—
Desai et al. 2022 (cohort 2) [18]		Observational study	35,004	Empagliflozin	12 months	17,502	72.15 ± 5.27	9,087	—	—	—
				GLP-1RA		17,502	72.13 ± 5.33	9,117	—	—	—
Butt et al. 2022 [19]	353 sites across 20 countries	Pre-specified analysis of the DELIVER Trial	6,258	Dapagliflozin vs. Placebo	8 months	Class 1 frailty (FI ≤0.210; i.e. not frail) = 2,354 (37.6%)	70.1 ± 10.3	1,308	—	54.2 ± 9.1	6.2 ± 1.2
						Class 2 frailty (FI 0.211–0.310; i.e. more frail) = 2,413 (38.6%)	72.6 ± 9.0	1,363	—	54.2 ± 8.8	6.6 ± 1.3
						Class 3 frailty (FI ≥0.311; i.e. most frail) = 1,491 (23.8%)	72.7 ± 8.8	841	—	54.1 ± 8.3	7.1 ± 1.6
Butt et al. 2022 [20]	410 sites in 20 countries	*Post hoc* analysis of the DAPA-HF trial	4,742	Dapagliflozin vs. Placebo	Median follow-up time = 18.2 months	Class 1 frailty (FI ≤0.210; i.e. not frail) = 2,392 (50.4%)	63.6 ± 11.6	1844	—	30.2 ± 7.0	Median 5.9 (range 5.6–6.4)
						Class 2 frailty (FI 0.211–0.310; i.e. more frail) = 1,606 (33.9%)	68.8 ± 9.4	1,225	—	31.9 ± 6.5	Median 6.2 (range 5.8–7.0)
						Class 3 frailty (FI ≥0.311; i.e. most frail) = 744 (15.7%)	69.8 ± 9.0	564	—	32.2 ± 6.3	Median 6.7 (range 6.0–7.7)

LVEF = left ventricular ejection fraction; GLP-1RA = glucagon-like peptide-1 receptor agonists; SD = standard deviation.

**Figure 2:**
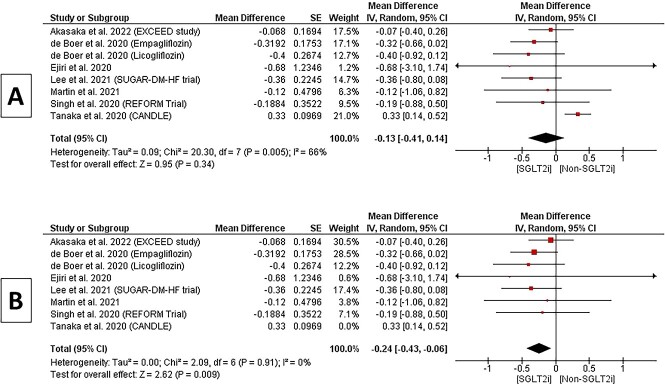
A and B Forest plot of the change in HbA1c%.

## Results

### Literature search results and study characteristics

A total of 5,362 records were initially identified through database searching, with an additional 27 articles identified through manual reference checks of relevant articles. After removing duplicates, 3,202 unique records were retrieved and were manually screened for the eligibility criteria. Finally, 20 studies (22 reports) fulfilled the eligibility criteria and were included in our study [40–60], with three articles included exclusively in the qualitative synthesis [40, 58, 59] (Figure 1). The included studies comprised 77,083 patients (56.96% males; *n* = 43,905) and assessed SGLT2 inhibitors compared with a placebo or any other glucose-lowering agent among the targeted population with HF and T2DM. Ten studies were RCTs [41, 42, 44, 46, 51–56], seven studies were observational [40, 43, 47–50, 57] and one study was a secondary analysis of the EMPEROR-Preserved trial [45]. In a prespecified analysis of the DELIVER trial by Butt et al. 2022 [59], the Rockwood cumulative deficit approach was used to assess frailty. Patients were classified into three groups based on their Frailty Index (FI) scores: FI class 1 [not frail] comprised those with a FI ≤0.210, FI class 2 [moderately frail] comprised those with a FI of 0.211–0.310, while FI class 3 [most frail] comprised those with a FI of ≥0.311. Class 1, class 2 and class 3 frailty were identified in 2354 (37.6%), 2,413 (38.6%) and 1,491 (23.8%). While in a *post hoc* analysis of the DAPA-HF trial (410 sites across 20 countries) [58], class 1, class 2 and class 3 frailty were identified in 2392 (50.4%), 1,606 (33.9%) and 744 (15.7%). The study characteristics and demographic data of the participants are presented in Table 1.

### Risk of bias assessment

Most studies showed a low risk in most Cochrane ROB tool domains. The summary of the ROB assessment of the included RCTs is shown in Figure S1 and Figure S2, while the ROB assessment of the included observational studies is presented in Table S1. The risk of bias assessment suggested that most of the data in this meta-analysis originated from studies with good evidence. We observed no significant evidence of publication bias in our study.

### Change in HbA1c%

Eight studies provided adequate data for this outcome. The pooled MD for % change of HbA1c did not show a statistically significant difference in SGLT2 inhibitors over the non-SGLT2 inhibitors arm (MD −0.13, 95% CI −0.41 to 0.14, *P =* 0.34). Heterogeneity within this subgroup was moderate (*P =* 0.005; *I*^2^ = 66%), Figure 2(a). However, in a sensitivity analysis excluding a study [2] based on lower dose of Canagliflozin, there was a statistically significant difference in HbA1c (MD −0.24, 95% CI −0.43 to −0.06, *P* = 0.009); the pooled studies were homogenous (*P =* 0.91; *I*^2^ = 0%), as shown in Figure 2 (b)***.*** Additional analysis, conducted in accordance with the study's design, yielded similar findings, as illustrated in Figure S3.

### Cardio-renal and safety outcomes

**Figure 3:**
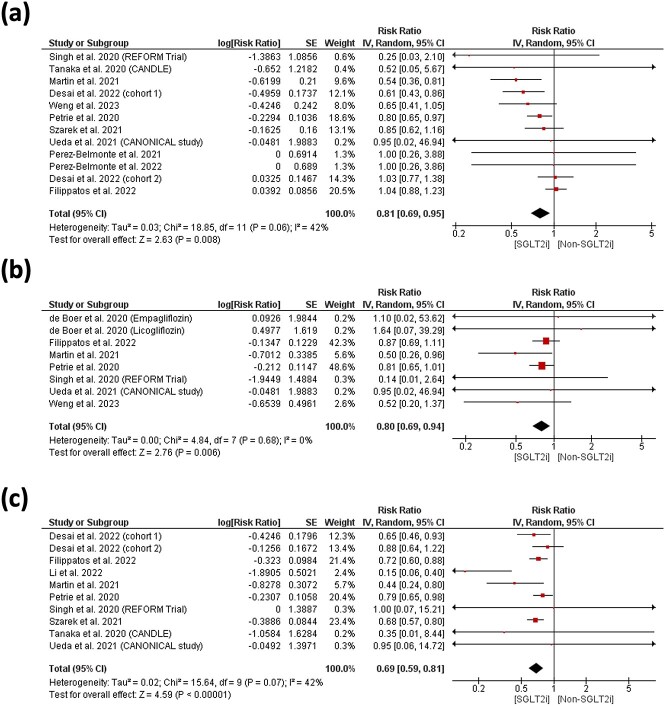
Forest plot of risk of (a) all-cause mortality, (b) cardiac death and (c) HHF

Overall, an RR of 0.81 (95% CI 0.69–0.95, *P =* 0.008) and 0.80 (0.69–0.94, *P =* 0.006) corresponding to a relative risk reduction of 19 and 20% in favour of SGLT2 inhibitors was found in terms of all-cause mortality and cardiac death, Figure 3(a) and (b). No statistically significant difference was found in all-cause mortality between SGLT2 inhibitor and control groups 0.90 (0.87–1.05) in RCT. However, observational studies indicated a significant reduction in mortality associated with SGLT2 inhibitors in real-world settings 0.81(0.69–0.95, *P* = 0.02), Figure S4. Further analysis demonstrated a consistent and statistically significant favouring of SGLT2 inhibitors in reducing the risk of cardiac death. This effect is particularly prominent in real-world observational studies, but it remains statistically significant when combined with RCT data, Figure S5.

Rate ratios for renal progression/composite renal endpoint and AKI did not show any difference 0.88 (0.65–1.13) and 0.92 (0.29–2.91), respectively, between the two groups. Overall, an RR of 0.69 (0.59–0.81, *P <* 0.00001) corresponding to a relative risk reduction of 31% in favour of SGLT2 inhibitors was found for HHF [the requirement of intravenous medications such as cardiotonics, diuretics and vasodilators to treat worsening HF] Figure 3(c). This benefit is particularly pronounced in RCTs and remains significant when considering both RCT and observational data, despite we observed a higher heterogeneity in observational studies, which may reflect real-world variations, Figure S6.

The overall RR however did not differ between both groups for worsening HF 0.55 (0.22–1.37), atrial fibrillation 1.67 (0.29–9.49) or DKA 0.94 (0.33–2.67). Regarding the incidence of thromboembolic events Acute coronary syndrome (ACS) or cerebral vascular occlusion, the overall RR did not favour either of the two groups 0.93 (0.76–1.13); pooled studies were homogenous (*P =* 0.71). The detailed safety profile is reported in Table 2 and Figure S7.

**Table 2:** Summary of the safety profile results

	Studies	RR (IV, Random, 95% CI)	*P*(Z)-value	*P*(Q) Hetero.; I-Sqr (Q)
Thromboembolic (ACS or Cerebral vascular occlusion)	8	0.93 [0.76, 1.13]	0.46	*P* = 0.71; *I*^2^ = 0%
All-cause mortality	**12**	**0.81 [0.69, 0.95]**	**0.008**	***P* = 0.06; *I*** ^**2**^ **= 42%**
Cardiac death	**8**	**0.80 [0.69, 0.94]**	**0.006**	***P* = 0.68; *I*** ^**2**^ **= 0%**
HHF	**10**	**0.69 [0.59, 0.81]**	**< 0.00001**	***P* = 0.07); *I*** ^**2**^ **= 42%**
Atrial fibrillation	3	1.67 [0.29, 9.49]	0.56	*P* = 0.86; *I*^2^ = 0%
Worsening HF	3	0.55 [0.22, 1.37]	0.2	*P* = 0.84; *I*^2^ = 0%
Renal progression/Composite renal end point	4	0.88 [0.69, 1.13]	0.31	*P* = 0.39; *I*^2^ = 1%
AKI	3	0.92 [0.29, 2.91]	0.89	*P* = 0.01; *I*^2^ = 77%
DKA	5	0.94 [0.33, 2.67]	0.91	*P* = 0.68; *I*^2^ = 0%

In Becher *et al.* 2021 [40], the authors documented a 30% reduction in the risk of CV death/first HHF among SGLT2 inhibitors users over a median follow-up of 256 days, with a hazard ratio of 0.70 and CI 0.52–0.95, and this was observed irrespective of Ejection Fraction (EF), prior metformin treatment or kidney function. Use of SGLT2 inhibitors was also linked to a decreased risk of (i) all-cause and CV death, (ii) CV hospitalisation and HHF and (iii) CV death/myocardial infarction/stroke.

### SGLT2 inhibitors and frailty

In the pre-specified analysis of the DELIVER trial [59], the impact of dapagliflozin was consistent among different levels of FI for worsening HF, HHF, CV death, all-cause mortality and the composite of total HF events and CV death. The HRs and RRs indicated a reduction in risk with dapagliflozin across FI classes. Specifically, the lowest to highest FI classes showed HRs of 0.87–0.69 for worsening HF, HRs of 0.83–0.69 for HF hospitalisation, HRs of 0.84–0.79 for CV death, HRs of 0.92–0.90 for all-cause mortality and RRs of 0.85–0.71 for the composite outcome.

Furthermore, the *post hoc* analysis of the DAPA-HF trial [58] confirmed the beneficial effects of dapagliflozin across different FI classes. The number needed to treat to prevent one event per 100 person-years decreased as FI class increased, indicating a greater efficacy of dapagliflozin in more frail patients. The HRs of composite and individual outcomes for worsening HF, hospitalisation or CV death showed consistent reductions across all FI classes compared with placebo arm. The magnitude of the reduction in these outcomes was generally more pronounced in the most fragile patients. Despite the consistent efficacy of dapagliflozin in reducing adverse outcomes, the study did not find any significant differences between dapagliflozin and placebo in terms of discontinuation of trial therapy or serious adverse events across all FI categories.

### Subgroup analysis

A subgroup analysis was performed on individuals 65 and older with varying SD to consider the age variation. In terms of cardiac death, individuals aged 65 with a lower SD group had a risk estimate of 0.68 (0.49–0.96), while those with a higher SD group had a risk estimate of 0.88 (0.69–0.94). All-cause mortality risk estimates were 0.72 (95% CI: 0.61, 0.85), compared with 0.91 (0.76–1.08) for the higher SD group. In terms of HF hospitalisation, the risk estimate for the lower SD group was 0.45 (0.22–0.91), compared with 0.71 (0.64–0.80) for the higher SD group. For the efficacy profile based on HbA1c levels, the difference for the lower SD group was −0.28 (−0.63 to 0.06), compared with −0.23 (−0.45 to −0.01) for the higher SD group, Figure S8**.**

## Discussion

Our study showed that SGLT2 inhibitors significantly reduced the risk of all-cause mortality, cardiac death and HHF in frail or older patients (aged ≥65 years) with T2DM and HF. While SGLT2 inhibitors did not substantially improve HbA1c levels overall (*P* = 0.34), a sensitivity analysis excluding a study with lower doses of Canagliflozin revealed a statistically significant reduction in HbA1c (*P* = 0.009). Interestingly, the *post hoc* analyses of the pivotal trials, DELIVER and DAPA-HF trials, confirmed that the positive effects of SGLT2 inhibitors on the CV adverse outcomes were consistent across the different levels of FI. The magnitude of the reduction in these outcomes was generally more pronounced in the most fragile patients [58, 59].

Frail and older patients with T2DM have consistently shown a progressive increase in the risk of adverse CV outcomes. Previous reports showed synergistic effects between frailty/ageing and T2DM, leading to higher risks of acute HF, coronary artery disease and stroke [61, 62]. In the presence of HF, older and frail patients with T2DM may have a greater risk of adverse CV outcomes [62–64]. While pivotal clinical trials and meta-analyses demonstrated the clinical benefits of SGLT2 inhibitors on HF-related adverse outcomes in patients with HFrEF [41, 50, 65], there is a lack of ample evidence to support the clinical benefits of SGLT2 inhibitors in the context of frail or older patients. To our knowledge, this is the first meta-analysis that evaluated the CV outcomes of SGLT2 inhibitors in this important patient group. Our findings align with previous meta-analysis that demonstrated a lower risk of HHF, CV death and all-cause mortality in T2DM patients with HF who received SGLT2 inhibitors [66, 67] and confirmed that the clinical benefits of SGLT2 inhibitors on CV outcomes extend to older and frail HF patients with T2DM.

Several mechanisms can potentially drive the positive effects of SGLT2 inhibitors in older or people with frailty with HF and T2DM. Growing evidence suggests that SGLT2 inhibitors improve the efficiency of myocardial metabolism by utilising ketone bodies as a primary substrate [68]. SGLT2 inhibitors have also been shown to reverse cardiac remodelling, attenuate inflammatory markers, [68], enhanced glucosuria and natriuresis, lowering blood pressure, improve hemodynamics, attenuate oxidative stress and decrease reperfusion injury [69]. In the context of HF, recent reports suggested that the SGLT2 inhibitors-mediated volume unloading and sustained improvement in the left ventricular filling pressure may contribute to the reduced risk of adverse CV outcomes, particularly HHF [70] although this mechanism was not supported by subanalysis of the EMPEROR-Reduced trial [71].

The main advantages of SGLT2 inhibitors usage in older frail patients are driven by a reduction in cardio-renal events (reduced CV mortality, HHF and renal adverse events) and improved HF-related health status [14, 16, 22, 72–74] rather than its glycaemic benefits. In the present meta-analysis, we observed comparable changes in the HbA1c between the SGLT2 inhibitors and non-SGLT2 inhibitors arms; this difference became significant (*P* = 0.009) in the sensitivity analysis, excluding TANAKA et al. 2020 (CANDLE) [54], which evaluated canagliflozin 100 mg, rather than the more effective 300 mg dose [75]. According to two meta-analyses, canagliflozin 300 mg was more effective in lowering HbA1c levels than either dapagliflozin or empagliflozin [76–78].

HF and T2DM are associated with a prothrombotic state, a leading cause of hospitalisation and death in HF patients, especially in older people [79] and renal dysfunction. Both conditions can interact to induce renal damage through multiple pathways. The present meta-analysis showed no significant difference in the incidence of thromboembolic events and renal progression/composite renal endpoint between patients with and without SGLT2 inhibitors. The exact reason for this is unclear but we speculate that the heterogeneity in the studied population and length of follow-up may represent potential explanations. It is also reassuring that the incidence of DKA was observed to be not significantly increased in the people with frailty and older people in our study. This is important due to the high risks of volume depletion, reduce food intake and risk of acute illness in this patient group, which might increase the risk of developing DKA with concurrent use of SGLT2 inhibitors.

When all the studies in which the mean age of the population was 65 years or above were considered, the analysis showed decreased risk of HF outcomes associated with SGLT2 inhibitors. This finding, however, should be interpreted with caution as the age data exhibited a wide SD, suggesting that the study cohorts lacked homogeneity. A subgroup analysis was therefore carried out in order to examine the influence of age, with particular emphasis on subjects who were older than 65 years. The data showed no statistical significance with respect to all-cause mortality and cardiac death, a finding that could be related to the few studies published on this issue and which exposes a gap in the literature.

Some limitations of our study needs to be highlighted; (i) the small number of studies included and restrictions to English language, (ii) a lack of standardisation of the interventions being investigated across the studies, (iii) the existence of residual confounding or selection bias cannot be ruled out, even though observational studies gather numerous variables that permit executing substantial adjustments utilising PS matching, (iv) it is conceivable that patients may have unmeasured traits linked with decreased risk, (v) due to the lack of studies and data, we could not perform a subgroup analysis based on the frailty status and (vi) we have not included other adverse outcomes such as urinary or genito-tract infection due to our focus on cardio-metabolic outcomes. Additionally, we acknowledge a potential limitation related to reporting bias, including language bias. We excluded non-English articles to maintain language consistency and prevent potential misinterpretations due to language nuances or translation errors. While this decision was made to ensure the accuracy of our analysis, it may have inadvertently restricted the inclusivity of our study, potentially introducing bias by omitting relevant non-English publications influenced by pharmaceutical interests. This limitation is prominently noted to emphasise its impact on the comprehensiveness of our research.

### Clinical implications and conclusions

The present meta-analysis confirmed that the cardioprotective advantages of SGLT2 inhibitors extend to the frail/older population, who are often underrepresented in clinical trials. Our findings provide further evidence to reinforce recommendations of initiating SGLT2 inhibitors early and not to miss the opportunity of reduced risks of adverse CV outcomes and avoid therapeutic inertia in this patient group. However, a multidisciplinary approach is crucial in the decision-making process for managing older individuals or people with frailty with T2DM and HF to effectively integrate SGLT2 inhibitors into treatment pathways [80]. In addition, co-morbidities, disability, frailty, anticipated life expectancy, patient preferences and therapeutic goals should all be considered while developing a treatment plan for this population.

## Supplementary Material

aa-23-1433-File002_afad254

## References

[1] Seferović PM , Petrie MC, Filippatos GS et al. Type 2 diabetes mellitus and heart failure: a position statement from the Heart Failure Association of the European Society of Cardiology. Eur J Heart Fail 2018; 20: 853–72. 29520964 10.1002/ejhf.1170

[2] Targher G , Dauriz M, Laroche C et al. In-hospital and 1-year mortality associated with diabetes in patients with acute heart failure: results from the ESC-HFA Heart Failure Long-Term Registry. Eur J Heart Fail 2017; 19: 54–65. 27790816 10.1002/ejhf.679

[3] Dauriz M , Targher G, Laroche C et al. Association between Diabetes and 1-year adverse clinical outcomes in a multinational cohort of ambulatory patients with chronic heart failure: results from the ESC-HFA Heart Failure Long-Term Registry. Diabetes Care 2017; 40: 671–8. 28255009 10.2337/dc16-2016

[4] Savarese G , Jonsson Å, Hallberg AC, Dahlström U, Edner M, Lund LH. Prevalence of, associations with, and prognostic role of anemia in heart failure across the ejection fraction spectrum. Int J Cardiol 2020; 298: 59–65. 31521440 10.1016/j.ijcard.2019.08.049

[5] Sinclair A , Saeedi P, Kaundal A, Karuranga S, Malanda B, Williams R. Diabetes and global ageing among 65-99-year-old adults: findings from the International Diabetes Federation Diabetes Atlas, 9(th) edition. Diabetes Res Clin Pract 2020; 162: 108078. 32068097 10.1016/j.diabres.2020.108078

[6] Sezgin D , O’Donovan M, Cornally N, Liew A, O’Caoimh R. Defining frailty for healthcare practice and research: a qualitative systematic review with thematic analysis. Int J Nurs Stud 2019; 92: 16–26. 30690163 10.1016/j.ijnurstu.2018.12.014

[7] Clegg A , Young J, Iliffe S, Rikkert MO, Rockwood K. Frailty in elderly people. Lancet 2013; 381: 752–62. 23395245 10.1016/S0140-6736(12)62167-9 PMC4098658

[8] Sinclair AJ , Abdelhafiz AH, Rodríguez-Mañas L. Frailty and sarcopenia - newly emerging and high impact complications of diabetes. J Diabetes Complications 2017; 31: 1465–73. 28669464 10.1016/j.jdiacomp.2017.05.003

[9] Hanlon P , Fauré I, Corcoran N et al. Frailty measurement, prevalence, incidence, and clinical implications in people with diabetes: a systematic review and study-level meta-analysis. Lancet Healthy Longev 2020; 1: e106–16. 33313578 10.1016/S2666-7568(20)30014-3 PMC7721684

[10] Sinclair AJ , Abdelhafiz A, Dunning T et al. An international position statement on the management of frailty in diabetes mellitus: summary of recommendations 2017. J Frailty Aging 2018; 7: 10–20. 29412437 10.14283/jfa.2017.39

[11] LeRoith D , Biessels GJ, Braithwaite SS et al. Treatment of diabetes in older adults: an Endocrine Society* clinical practice guideline. J Clin Endocrinol Metab 2019; 104: 1520–74. 30903688 10.1210/jc.2019-00198 PMC7271968

[12] Ni L , Yuan C, Chen G, Zhang C, Wu X. SGLT2i: beyond the glucose-lowering effect. Cardiovasc Diabetol 2020; 19: 98. 32590982 10.1186/s12933-020-01071-y PMC7320582

[13] Zinman B , Lachin JM, Inzucchi SE. Empagliflozin, cardiovascular outcomes, and mortality in type 2 diabetes. N Engl J Med 2016; 374: 1094. 10.1056/NEJMc1600827 26981940

[14] Heerspink HJ , Perkins BA, Fitchett DH, Husain M, Cherney DZI. Sodium glucose cotransporter 2 inhibitors in the treatment of diabetes mellitus: cardiovascular and kidney effects, potential mechanisms, and clinical applications. Circulation 2016; 134: 752–72. 27470878 10.1161/CIRCULATIONAHA.116.021887

[15] Neal B , Perkovic V, Matthews DR. Canagliflozin and cardiovascular and renal events in type 2 diabetes. N Engl J Med 2017; 377: 2099. 29166232 10.1056/NEJMc1712572

[16] Wiviott SD , Raz I, Bonaca MP et al. Dapagliflozin and cardiovascular outcomes in type 2 diabetes. N Engl J Med 2018; 380: 347–57. 30415602 10.1056/NEJMoa1812389

[17] Cavender MA , Norhammar A, Birkeland KI et al. SGLT-2 inhibitors and cardiovascular risk: an analysis of CVD-REAL. J Am Coll Cardiol 2018; 71: 2497–506. 29852973 10.1016/j.jacc.2018.01.085

[18] Anker SD , Butler J, Filippatos G et al. Empagliflozin in heart failure with a preserved ejection fraction. N Engl J Med 2021; 385: 1451–61. 34449189 10.1056/NEJMoa2107038

[19] Mahaffey KW , Jardine MJ, Bompoint S et al. Canagliflozin and cardiovascular and renal outcomes in type 2 diabetes mellitus and chronic kidney disease in primary and secondary cardiovascular prevention groups. Circulation 2019; 140: 739–50. 31291786 10.1161/CIRCULATIONAHA.119.042007 PMC6727954

[20] Perkovic V , Jardine MJ, Neal B et al. Canagliflozin and renal outcomes in type 2 diabetes and nephropathy. N Engl J Med 2019; 380: 2295–306. 30990260 10.1056/NEJMoa1811744

[21] Kosiborod M , Lam CSP, Kohsaka S et al. Cardiovascular events associated with SGLT-2 inhibitors versus other glucose-lowering drugs: the CVD-REAL 2 study. J Am Coll Cardiol 2018; 71: 2628–39. 29540325 10.1016/j.jacc.2018.03.009

[22] Packer M , Anker SD, Butler J et al. Cardiovascular and renal outcomes with empagliflozin in heart failure. N Engl J Med 2020; 383: 1413–24. 32865377 10.1056/NEJMoa2022190

[23] Solomon SD , de Boer RA, DeMets D et al. Dapagliflozin in heart failure with preserved and mildly reduced ejection fraction: rationale and design of the DELIVER trial. Eur J Heart Fail 2021; 23: 1217–25. 34051124 10.1002/ejhf.2249 PMC8361994

[24] Bhatt DL , Szarek M, Steg PG et al. Sotagliflozin in patients with diabetes and recent worsening heart failure. N Engl J Med 2021; 384: 117–28. 33200892 10.1056/NEJMoa2030183

[25] Pollack R , Cahn A. SGLT2 inhibitors and safety in older patients. Heart Fail Clin 2022; 18: 635–43. 36216492 10.1016/j.hfc.2022.03.002

[26] Liu Y , An C, Liu P, Yang F, Zhao Q. Comparative safety of sodium-glucose co-transporter 2 inhibitors in elderly patients with type 2 diabetes mellitus and diabetic kidney disease: a systematic review and meta-analysis. Ren Fail 2023; 45: 2217287. 37246403 10.1080/0886022X.2023.2217287 PMC10228313

[27] Hahn K , Ejaz AA, Kanbay M, Lanaspa MA, Johnson RJ. Acute kidney injury from SGLT2 inhibitors: potential mechanisms. Nat Rev Nephrol 2016; 12: 711–2. 27847389 10.1038/nrneph.2016.159

[28] Szalat A , Perlman A, Muszkat M, Khamaisi M, Abassi Z, Heyman SN. Can SGLT2 inhibitors cause acute renal failure? Plausible role for altered glomerular hemodynamics and medullary hypoxia. Drug Saf 2018; 41: 239–52. 28952138 10.1007/s40264-017-0602-6

[29] O'Neill J , Fasching A, Pihl L, Patinha D, Franzén S, Palm F. Acute SGLT inhibition normalizes O2 tension in the renal cortex but causes hypoxia in the renal medulla in anaesthetized control and diabetic rats. Am J Physiol Renal Physiol 2015; 309: F227–34. 26041448 10.1152/ajprenal.00689.2014

[30] Type 2, *diabetes in adults: management-NICE guideline [NG28]*. 2015 Last updated 29th June 2022.

[31] Strain WD , Down S, Brown P, Puttanna A, Sinclair A. Diabetes and frailty: an expert consensus statement on the management of older adults with type 2 diabetes. Diabetes Ther 2021; 12: 1227–47. 33830409 10.1007/s13300-021-01035-9 PMC8099963

[32] Page MJ , McKenzie JE, Bossuyt PM et al. The PRISMA 2020 statement: an updated guideline for reporting systematic reviews Declaración PRISMA 2020: una guía actualizada para la publicación de revisiones sistemáticas*.* Revista Panamericana de Salud Publica= Pan. *Rev Panam Salud Publica*. Pan Am J Public Health 2022; 46: e112. 10.1186/s13643-021-01626-4 PMC8008539 33781348

[33] Higgins JP , Thomas J, Chandler J et al. Cochrane Handbook for Systematic Reviews of Interventions. John Wiley & Sons, Chichester, UK; 2019.

[34] Higgins JP , Altman DG. Assessing risk of bias in included studies. In: Cochrane Handbook for Systematic Reviews of Interventions. The Cochrane collaboration, 2008; 187–241.

[35] Wells G , Shea B, O'Connell D et al. The Newcastle-Ottawa Scale (NOS) for Assessing the Quality of Nonrandomised Studies in Meta-Analyses Ottawa. Ontario, Canada: Ottawa Hospital Research Institute, 2000.

[36] Ebada MA , Elmatboly AM, Ali AS et al. An updated systematic review and meta-analysis about the safety and efficacy of infliximab biosimilar, CT-P13, for patients with inflammatory bowel disease. Int J Colorectal Dis 2019; 34: 1633–52. 31492986 10.1007/s00384-019-03354-7

[37] Higgins JPT, Thomas J, Chandler J, Cumpston M, Li T, Page MJ, Welch VA (eds). Cochrane Handbook for Systematic Reviews of Interventions version 6.4 (updated August 2023). Cochrane, 2023.

[38] Borenstein M , Hedges LV, Higgins JPT, Rothstein HR. A basic introduction to fixed-effect and random-effects models for meta-analysis. Res Synth Methods 2010; 1: 97–111. 26061376 10.1002/jrsm.12

[39] Higgins JP , Thompson SG, Deeks JJ, Altman DG. Measuring inconsistency in meta-analyses. BMJ 2003; 327: 557–60. 12958120 10.1136/bmj.327.7414.557 PMC192859

[40] Becher PM , Schrage B, Ferrannini G et al. Use of sodium-glucose co-transporter 2 inhibitors in patients with heart failure and type 2 diabetes mellitus: data from the Swedish Heart Failure Registry. Eur J Heart Fail 2021; 23: 1012–22. 33599357 10.1002/ejhf.2131

[41] Akasaka H , Sugimoto K, Shintani A et al. Effects of ipragliflozin on left ventricular diastolic function in patients with type 2 diabetes and heart failure with preserved ejection fraction: the EXCEED randomized controlled multicenter study. Geriatr Gerontol Int 2022; 22: 298–304. 35212104 10.1111/ggi.14363 PMC9305927

[42] de Boer RA , Núñez J, Kozlovski P, Wang Y, Proot P, Keefe D. Effects of the dual sodium–glucose linked transporter inhibitor, licogliflozin vs placebo or empagliflozin in patients with type 2 diabetes and heart failure. Br J Clin Pharmacol 2020; 86: 1346–56. 32068914 10.1111/bcp.14248 PMC7318993

[43] Weng YF , Chen CY, Hwang SJ, Huang YB. Evaluation of sodium-glucose cotransporter 2 inhibitors for renal prognosis and mortality in diabetes patients with heart failure on diuretics. Kaohsiung J Med Sci 2023; 39: 416–25. 36625282 10.1002/kjm2.12635 PMC11895888

[44] Ejiri K , Miyoshi T, Kihara H et al. Effects of luseogliflozin and voglibose on high-risk lipid profiles and inflammatory markers in diabetes patients with heart failure. Sci Rep 2022; 12: 15449. 36104378 10.1038/s41598-022-19371-6 PMC9474821

[45] Filippatos G , Butler J, Farmakis D et al. Empagliflozin for heart failure with preserved left ventricular ejection fraction with and without diabetes. Circulation 2022; 146: 676–86. 35762322 10.1161/CIRCULATIONAHA.122.059785 PMC9422757

[46] Lee MM , Brooksbank KJM, Wetherall K et al. Effect of empagliflozin on left ventricular volumes in patients with type 2 diabetes, or prediabetes, and heart failure with reduced ejection fraction (SUGAR-DM-HF). Circulation 2021; 143: 516–25. 33186500 10.1161/CIRCULATIONAHA.120.052186 PMC7864599

[47] Li W , Katamreddy A, Kataria R, Myerson ML, Taub CC. Sodium-Glucose cotransporter-2 inhibitor use is associated with a reduced risk of heart failure hospitalization in patients with heart failure with preserved ejection fraction and type 2 diabetes mellitus: a real-world study on a diverse urban population. Drugs-Real World Outcomes 2021; 9: 53–62. 34478119 10.1007/s40801-021-00277-0 PMC8844327

[48] Martín E , López-Aguilera J, González-Manzanares R et al. Impact of canagliflozin in patients with type 2 diabetes after hospitalization for acute heart failure: a cohort study. J Clin Med 2021; 10: 505. 33535424 10.3390/jcm10030505 PMC7867051

[49] Pérez-Belmonte LM , Ricci M, Sanz-Cánovas J et al. Efficacy and safety of empagliflozin continuation in patients with type 2 diabetes hospitalised for acute decompensated heart failure. J Clin Med 2021; 10: 3540. 34441835 10.3390/jcm10163540 PMC8396978

[50] Pérez-Belmonte LM , Sanz-Cánovas J, Millán-Gómez M et al. Clinical benefits of empagliflozin in very old patients with type 2 diabetes hospitalized for acute heart failure. J Am Geriatr Soc 2022; 70: 862–71. 34843628 10.1111/jgs.17585

[51] Petrie MC , Verma S, Docherty KF et al. Effect of dapagliflozin on worsening heart failure and cardiovascular death in patients with heart failure with and without diabetes. JAMA 2020; 323: 1353–68. 32219386 10.1001/jama.2020.1906 PMC7157181

[52] Singh JS , Mordi IR, Vickneson K et al. Dapagliflozin versus placebo on left ventricular remodeling in patients with diabetes and heart failure: the REFORM trial. Diabetes Care 2020; 43: 1356–9. 32245746 10.2337/dc19-2187 PMC7245350

[53] Tamaki S , Yamada T, Watanabe T et al. Effect of empagliflozin as an add-on therapy on decongestion and renal function in patients with diabetes hospitalized for acute decompensated heart failure: a prospective randomized controlled study. Circ Heart Fail 2021; 14: e007048. 33663235 10.1161/CIRCHEARTFAILURE.120.007048

[54] Tanaka A , Hisauchi I, Taguchi I et al. Effects of canagliflozin in patients with type 2 diabetes and chronic heart failure: a randomized trial (CANDLE). ESC Heart Fail 2020; 7: 1585–94. 32349193 10.1002/ehf2.12707 PMC7373938

[55] Ueda T , Kasama S, Yamamoto M et al. Effect of the sodium-glucose cotransporter 2 inhibitor canagliflozin for heart failure with preserved ejection fraction in patients with type 2 diabetes. Circ Rep 2021; 3: 440–8. 34414333 10.1253/circrep.CR-21-0030 PMC8338435

[56] Szarek M , Bhatt DL, Steg PG et al. Effect of sotagliflozin on total hospitalizations in patients with type 2 diabetes and worsening heart failure: a randomized trial. Ann Intern Med 2021; 174: 1065–72. 34152828 10.7326/M21-0651

[57] Desai RJ , Glynn RJ, Everett BM et al. Comparative effectiveness of Empagliflozin in reducing the burden of recurrent cardiovascular hospitalizations among older adults with diabetes in routine clinical care. Am Heart J 2022; 254: 203–15. 36150454 10.1016/j.ahj.2022.09.008

[58] Butt JH , Dewan P, Merkely B et al. Efficacy and safety of dapagliflozin according to frailty in heart failure with reduced ejection fraction: a post hoc analysis of the DAPA-HF trial. Ann Intern Med 2022; 175: 820–30. 35467935 10.7326/M21-4776

[59] Butt JH , Jhund PS, Belohlávek J et al. Efficacy and safety of dapagliflozin according to frailty in patients with heart failure: a prespecified analysis of the DELIVER trial. Circulation 2022; 146: 1210–24. 36029465 10.1161/CIRCULATIONAHA.122.061754 PMC9815819

[60] Greene SJ , Vaduganathan M, Khan MS et al. Prevalent and incident heart failure in cardiovascular outcome trials of patients with type 2 diabetes. J Am Coll Cardiol 2018; 71: 1379–90. 29534825 10.1016/j.jacc.2018.01.047 PMC5832063

[61] Halter JB , Musi N, McFarland Horne F et al. Diabetes and cardiovascular disease in older adults: current status and future directions. Diabetes 2014; 63: 2578–89. 25060886 10.2337/db14-0020 PMC4113072

[62] Sinclair AJ , Abdelhafiz AH. Multimorbidity, frailty and diabetes in older people–identifying interrelationships and outcomes. J Pers Med 2022; 12: 1911. 36422087 10.3390/jpm12111911 PMC9695437

[63] Pandey A , Kitzman D, Reeves G. Frailty is intertwined with heart failure: mechanisms, prevalence, prognosis, assessment, and management. JACC Heart Fail 2019; 7: 1001–11. 31779921 10.1016/j.jchf.2019.10.005 PMC7098068

[64] Vitale C , Jankowska E, Hill L et al. Heart Failure Association of the European Society of Cardiology position paper on frailty in patients with heart failure. Eur J Heart Fail 2019; 21: 1299–305. 31646718 10.1002/ejhf.1611

[65] Kato ET , Silverman MG, Mosenzon O et al. Effect of dapagliflozin on heart failure and mortality in type 2 diabetes mellitus. Circulation 2019; 139: 2528–36. 30882238 10.1161/CIRCULATIONAHA.119.040130

[66] Zhao Z , Jin P, Zhang Y, Hu X, Tian C, Liu D. SGLT2 inhibitors in diabetic patients with cardiovascular disease or at high cardiovascular risk: a systematic review and meta-analysis of randomized controlled trials. Front Cardiovasc Med 2022; 9: 9. 10.3389/fcvm.2022.826684 PMC9087280 35557542

[67] Singh AK , Singh R. Cardiovascular outcomes with SGLT-2 inhibitors in patients with heart failure with or without type 2 diabetes: a systematic review and meta-analysis of randomized controlled trials. Diabetes Metab Syndr Clin Res Rev 2021; 15: 351–9. 10.1016/j.dsx.2021.01.006 33503584

[68] Saucedo-Orozco H , Voorrips SN, Yurista SR, de Boer RA, Westenbrink BD. Sglt2 inhibitors and ketone metabolism in heart failure. J Lipid Atheroscler 2022; 11: 1–19. 35118019 10.12997/jla.2022.11.1.1 PMC8792821

[69] Lopaschuk GD , Verma S. Mechanisms of cardiovascular benefits of sodium glucose co-transporter 2 (SGLT2) inhibitors: a state-of-the-art review. JACC Basic Transl Sci 2020; 5: 632–44. 32613148 10.1016/j.jacbts.2020.02.004 PMC7315190

[70] Rau M , Thiele K, Hartmann NUK et al. Empagliflozin does not change cardiac index nor systemic vascular resistance but rapidly improves left ventricular filling pressure in patients with type 2 diabetes: a randomized controlled study. Cardiovasc Diabetol 2021; 20: 1–12. 33413355 10.1186/s12933-020-01175-5 PMC7791833

[71] Packer M , Anker SD, Butler J et al. Empagliflozin in patients with heart failure, reduced ejection fraction, and volume overload: EMPEROR-reduced trial. J Am Coll Cardiol 2021; 77: 1381–92. 33736819 10.1016/j.jacc.2021.01.033

[72] Anker SD , Butler J, Filippatos GS et al. Evaluation of the effects of sodium–glucose co-transporter 2 inhibition with empagliflozin on morbidity and mortality in patients with chronic heart failure and a preserved ejection fraction: rationale for and design of the EMPEROR-Preserved Trial. Eur J Heart Fail 2019; 21: 1279–87. 31523904 10.1002/ejhf.1596

[73] McMurray JJ , Solomon SD, Inzucchi SE et al. Dapagliflozin in patients with heart failure and reduced ejection fraction. N Engl J Med 2019; 381: 1995–2008. 31535829 10.1056/NEJMoa1911303

[74] Zinman B , Wanner C, Lachin JM et al. Empagliflozin, cardiovascular outcomes, and mortality in type 2 diabetes. N Engl J Med 2015; 373: 2117–28. 26378978 10.1056/NEJMoa1504720

[75] Cefalu WT , Leiter LA, Yoon KH et al. Efficacy and safety of canagliflozin versus glimepiride in patients with type 2 diabetes inadequately controlled with metformin (CANTATA-SU): 52 week results from a randomised, double-blind, phase 3 non-inferiority trial. Lancet 2013; 382: 941–50. 23850055 10.1016/S0140-6736(13)60683-2

[76] Johnston R , Uthman O, Cummins E et al. Canagliflozin, dapagliflozin and empagliflozin monotherapy for treating type 2 diabetes: systematic review and economic evaluation. Health Technol Assess 2017; 21: 1–218. 10.3310/hta21020 PMC5292646 28105986

[77] Zaccardi F , Webb DR, Htike ZZ, Youssef D, Khunti K, Davies MJ. Efficacy and safety of sodium-glucose co-transporter-2 inhibitors in type 2 diabetes mellitus: systematic review and network meta-analysis. Diabetes Obes Metab 2016; 18: 783–94. 27059700 10.1111/dom.12670

[78] Stein P , Berg JK, Morrow L et al. Canagliflozin, a sodium glucose co-transporter 2 inhibitor, reduces post-meal glucose excursion in patients with type 2 diabetes by a non-renal mechanism: results of a randomized trial. Metabolism 2014; 63: 1296–303. 25110280 10.1016/j.metabol.2014.07.003

[79] Camm AJ , Sabbour H, Schnell O, Summaria F, Verma A. Managing thrombotic risk in patients with diabetes. Cardiovasc Diabetol 2022; 21: 160. 35996159 10.1186/s12933-022-01581-x PMC9396895

[80] Evans M , Morgan AR, Davies S, Beba H, Strain WD. The role of sodium-glucose co-transporter-2 inhibitors in frail older adults with or without type 2 diabetes mellitus. Age Ageing 2022; 51: afac201. 36201329 10.1093/ageing/afac201 PMC9536439

