# Supplementary material for: Sodium-glucose cotransporter-2 inhibitors (SGLT2) in frail or older people with type 2 diabetes and heart failure: a systematic review and meta-analysis
Source: Age Ageing. 2024 Jan 29;53(1):afad254. doi: 10.1093/ageing/afad254 (PMC10825241; doi:10.1093/ageing/afad254)

**Sodium-glucose Cotransporter-2 Inhibitors (SGLT2) in Frail or Older People with Type 2 Diabetes and Heart Failure: A Systematic Review and Meta-Analysis**

**Supplementary Data**

**Appendix table 1.** The Newcastle-Ottawa Scale (NOS) quality assessment of the included observational studies

**Fig. S1.** The risk of bias graph of all included RCTs.

**Fig. S2.** The risk of bias summary of all included RCTs.

**Fig. S3.** Forest plot analyzing HbA1c% change – subgroup analysis by study design

**Fig. S4.** Forest plot analyzing all-cause mortality – subgroup analysis by study design

**Fig. S5.** Forest plot analyzing cardiac death – subgroup analysis by study design**.**

**Fig. S6.** Forest plot analyzing hospitalization for heart failure– subgroup analysis by study design.

**Fig. S7.** Forest plots of some safety profile results

**Fig. S8.** Subgroup analysis

**Fig. S9.** Funnel plots

**Table S1. The Newcastle-Ottawa Scale (NOS) quality assessment of the included observatinal studies**

| Study | Selection of cohorts | | | | Comparability of cohorts | Outcome | | |
| --- | --- | --- | --- | --- | --- | --- | --- | --- |
|  | Representativeness of the exposed cohort | Selection of the non-exposed cohort | Ascertainment of exposure | Demonstration that outcome of interest was not present at start of study | Comparability of cohorts on the basis of the design or analysis | Assessment of outcome | Was follow up long enough for outcomes to occur | Adequacy of follow up of cohorts |
| Weng et al. 2023 | * | * | * | * | * |  | * | * |
| Becher et al. 2021 | * | * |  | * | * | * | * | * |
| Li et al. 2022 |  | * | * | * | * | * | * | * |
| Martin et al. 2021 |  | * | * | * | * | * | * | * |
| Perez-Belmonte et al. 2021 | * | * | * | * | * | * | * | * |
| Perez-Belmonte et al. 2022 | * | * | * | * | * | * | * | * |
| Desai et al. 2022 | * | * | * | * | * | * | * | * |

***Fig. S1. The risk of bias graph of all included RCTs.***
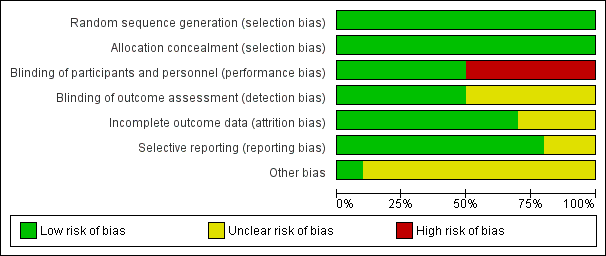


***Fig. S2. The risk of bias summary of all included RCTs.***


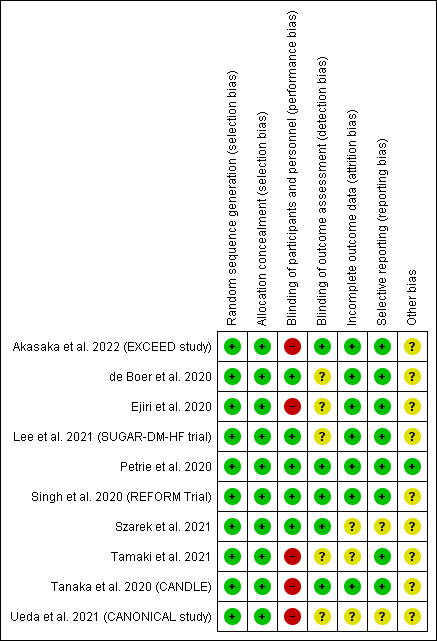


***Fig. S3. Forest plot analyzing HbA1c% change – subgroup analysis by study design***

**
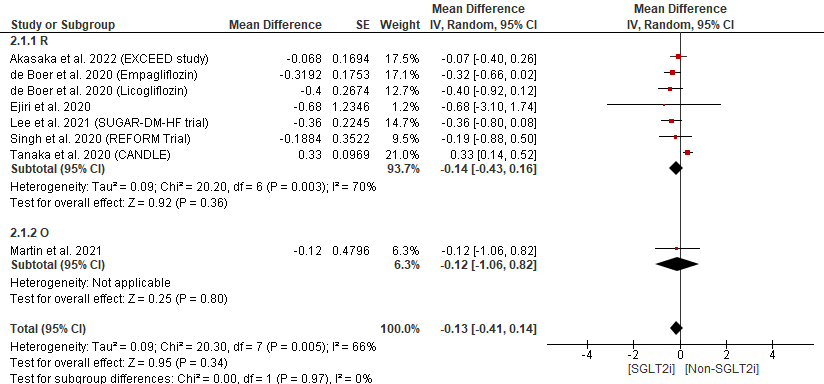
**

***Fig. S4. Forest plot analyzing all-cause mortality – subgroup analysis by study design***

**
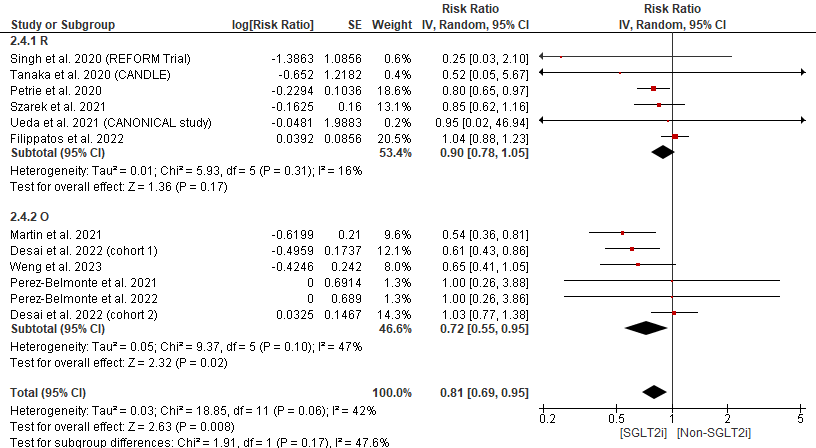
**

***Fig. S5. Forest plot analyzing cardiac death – subgroup analysis by study design.***

**
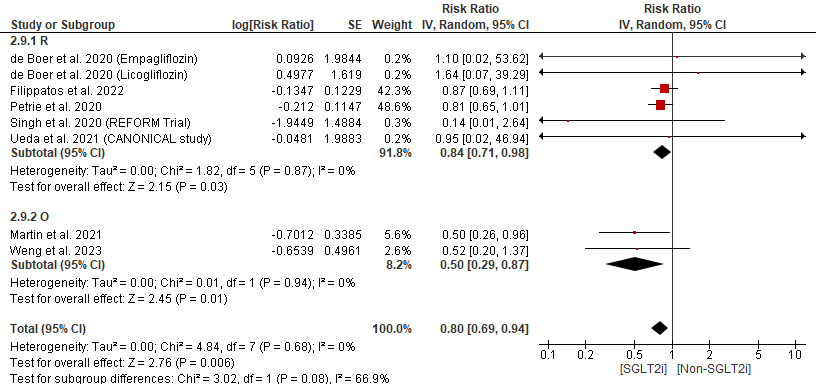
**

***Fig. S6. Forest plot analyzing hospitalization for heart failure– subgroup analysis by study design.***

**
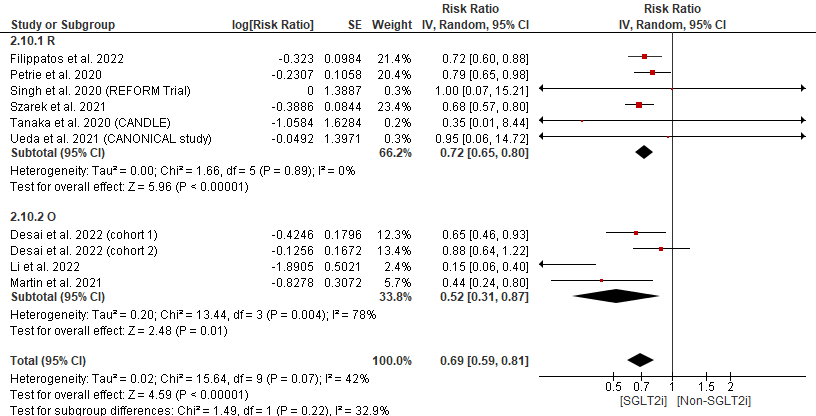
**

***Fig. S7. Forest plots of some safety profile results
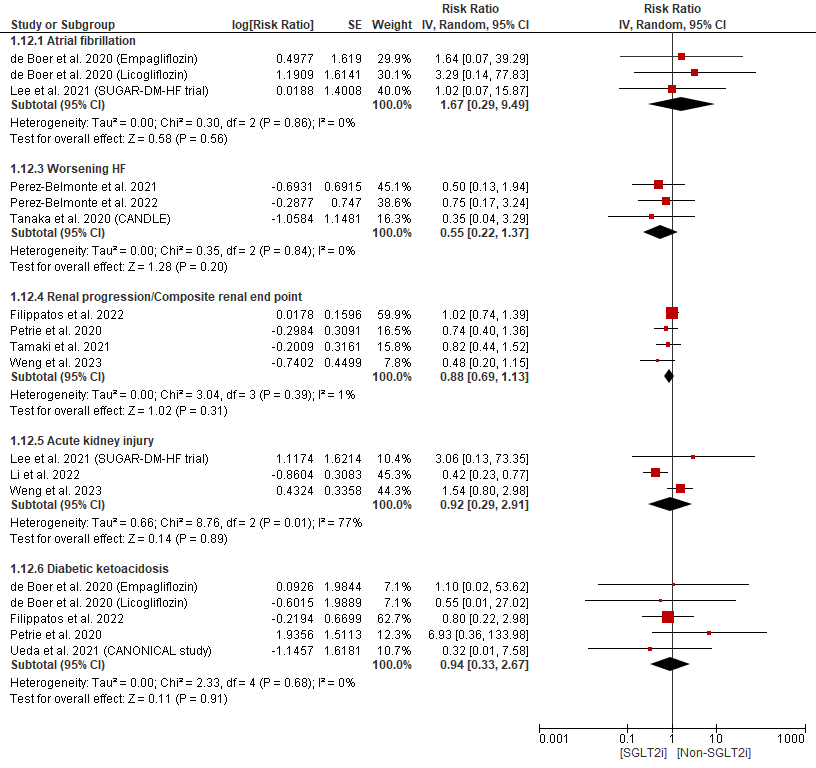
***

***Fig. S8. Subgroup analysis* accordance on individuals with 65 and older with varying Standard deviation**

***All cause-mortality***

***
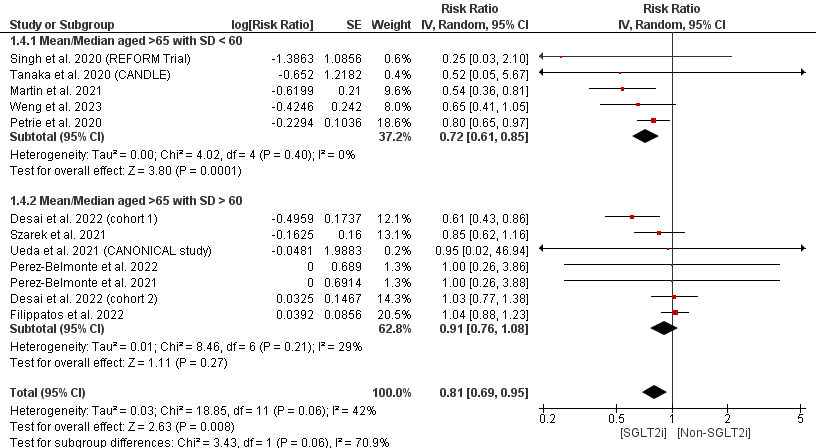
***

***Cardiac death***

***
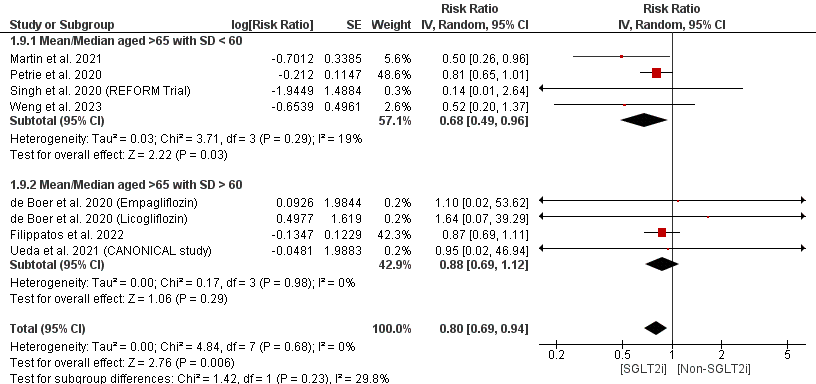
***

***Change in HbA1c***

***
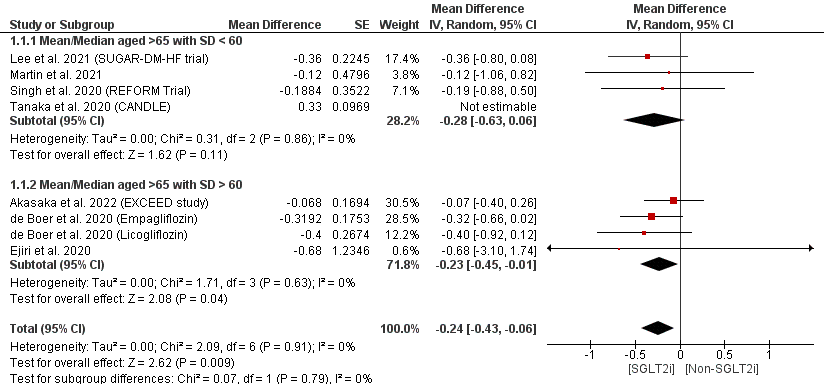
***

***Hospitalization op Heart Failure***

***
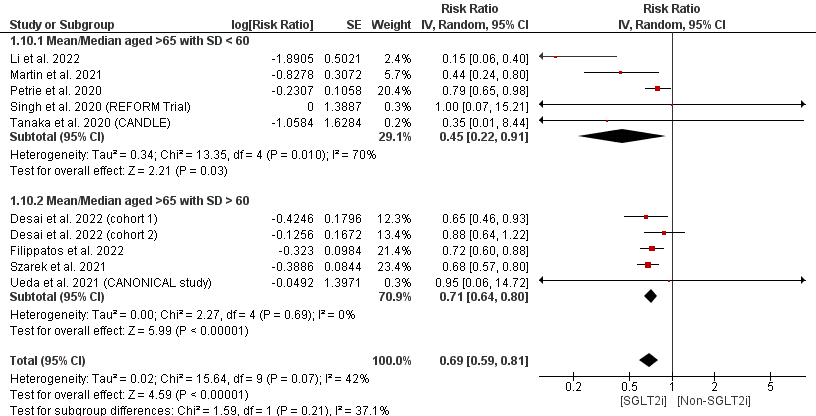
***

***Fig. S9. Funnel plots***


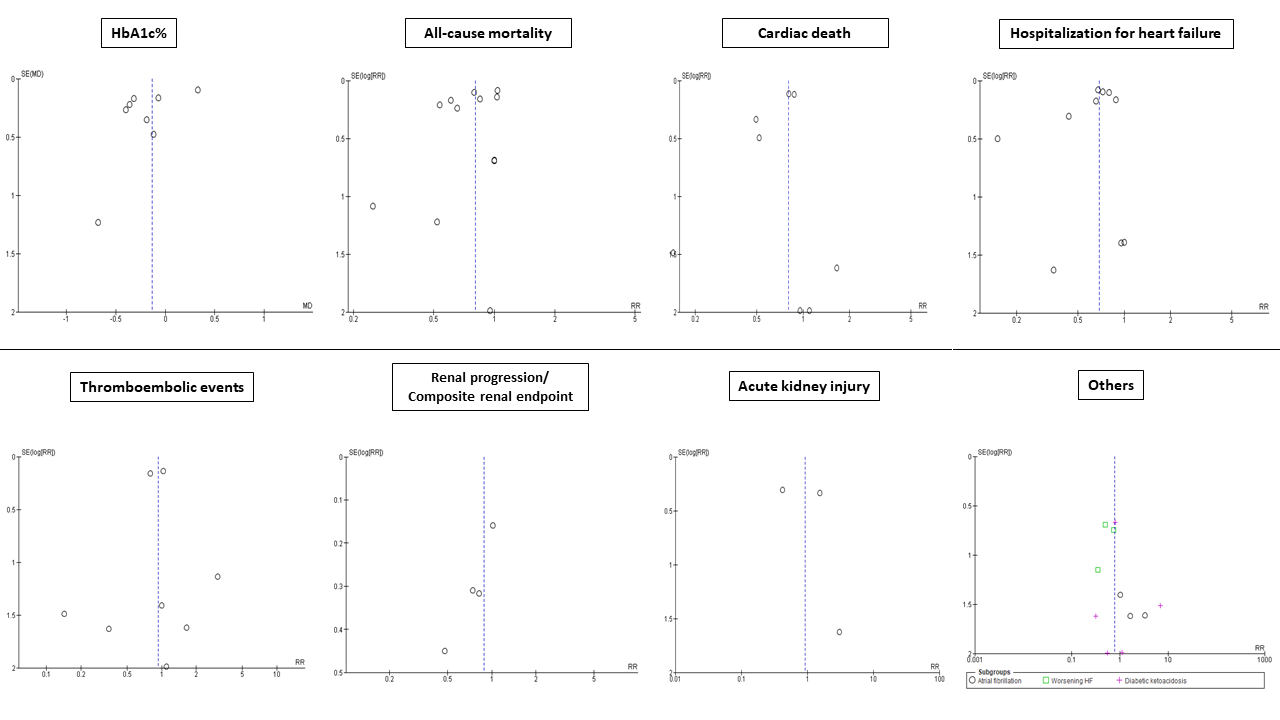

Supplement: aa-23-1433-File002_afad254 [file aa-23-1433-file002_afad254.docx]
